# Incidence of tolerance in children undergoing repeated administration of propofol for proton radiation therapy: a retrospective study

**DOI:** 10.1186/s12871-018-0587-4

**Published:** 2018-09-07

**Authors:** RyungA Kang, Byung Seop Shin, Young Hee Shin, Nam-Su Gil, Ye Na Oh, Ji Seon Jeong

**Affiliations:** 1 Department of Anesthesiology and Pain Medicine, Samsung Medical Center, Sungkyunkwan University school of Medicine, 81 Irwon-ro, Gangnam, Seoul, 06352 South Korea; 2 Department of Anesthesiology and Pain Medicine, Kangwon National University School of Medicine, Chuncheon, South Korea

**Keywords:** Propofol, Tolerance, Proton radiation therapy, Children, Deep sedation, Anesthesia

## Abstract

**Background:**

Propofol is an excellent hypnotic drug for use in repeated radiation procedures in young children. To date, tolerance to propofol generally does not develop in pediatric patients undergoing radiation therapy. However, several studies have suggested that there may be potential for development of tolerance to propofol. The aim of this study was to evaluate the development of a tolerance to propofol used for repeated deep sedation in children undergoing proton radiation therapy (PRT).

**Methods:**

All children undergoing PRT at our institution between December 2015 and January 2018 were eligible for inclusion in this study. Sedation was induced by a bolus dose of propofol (2.0 mg.kg^− 1^) followed by a continuous infusion of 250 μg.kg^− 1^.min^− 1^ via an infusion pump to achieve deep sedation. Sedation was maintained with the propofol infusion of 200 μg.kg^− 1^.min^− 1^, which was adjusted in 25 μg.kg^− 1^.min^− 1^ increments up or down as necessary to ensure deep sedation. The primary outcome was mean doses of propofol over time.

**Results:**

Fifty-eight children were analyzed. The mean (SD) age was 4.5 (2.1) years. The mean (SD) number of treatment sessions was 19 (7). Fifteen patients (26%) developed tolerance to propofol. However, there were no significant differences between the children who developed tolerance and the children who did not develop tolerance in mean propofol dose and awakening time over time (*p* = 0.887 and *P* = 0.652, respectively). Age, the number of PRT, and attending anesthesiologists was not significantly associated with the incidence of tolerance to propofol.

**Conclusion:**

Repeated prolonged deep sedation for PRT elicited multiple times over several weeks in young children using propofol did not develop tolerance in 74% of patients. Although the incidence of 26% tolerance to propofol may still be present, the increase in propofol dose was minimal. Therefore, the use of repeated propofol for children was safe.

## Background

Proton radiation therapy (PRT), which destroys abnormal tissues in the body via delicate control of the range of a proton beam, requires deep sedation in children to avoid movement and maintain a precise position during irradiation. [1–3] Since the total duration of treatment is several weeks, children undergoing PRT are repeatedly exposed to anesthetic agents. Repeated exposure to some anesthetic drugs can lead to the development of drug tolerance, which is defined as a decrease in a drug’s effect over time or the need to increase the drug’s dose over time to achieve the same effect. [4]

Propofol is an excellent hypnotic drug for use in repeated radiation procedures in young children because of its unique pharmacologic properties, such as rapid onset, fast recovery from anesthesia, and low incidence of nausea and vomiting. [5] To date, tolerance to propofol generally does not develop in pediatric patients undergoing radiation therapy. However, several studies have suggested that there may be potential for development of tolerance to propofol, either by increased clearance of the drug [6, 7] or by changes in the body’s sensitivity to the drug’s effects. [8, 9] Taken together, the data on tolerance to propofol seem to be controversial. Therefore, in this retrospective study, we aimed to evaluate the tolerance to propofol for repeated deep sedation in children undergoing PRT.

## Methods

All children aged 16 years or younger who underwent deep sedation for PRT at our institution (Samsung Medical Center in Seoul, Korea) between December 2015 and January 2018 were eligible for inclusion in this study. Among them, children who underwent only a simulation session and did not experience sedation with anesthetic agents were excluded. The Institutional Review Board of Samsung Medical Center approved this retrospective study (SMC 2018–02-121) and waived the requirement for written informed consent. All data were retrospectively collected from the computerized medical records. The collected data included patient baseline characteristics; site of irradiation; total number of treatments; total amount of propofol required; hemodynamic parameters including mean arterial blood pressure, heart rate, respiratory rate, and oxygen saturation; and the patient’s recovery profile in the post-anesthesia care unit (PACU).

The sedation procedure for radiation therapy was performed daily, with the exception of weekends or holidays, according to our standardized institutional protocol. Each session can last between 30 and 90 min depending on the complexity of the treatment set-up and delivery. Patients came to the treatment room having fasted according to the current American Society of Anesthesiologists guidelines and did not receive premedication before induction. All patients had a permanent pre-existing indwelling central venous catheter. Before induction, we administered a bolus of intravenous fluid (10 ml.kg^− 1^) to decrease the incidence of hypotension. [10] Standard monitoring including electrocardiogram, non-invasive arterial blood pressure measurement, pulse oximetry, and a facial mask for delivering supplemental oxygen and for end-tidal CO_2_ monitoring were installed and were continuously monitored in three-minutes intervals during radiation therapy. Prior to the PRT, we performed airway optimization in all patients. Sedation was induced by a bolus dose of propofol (2.0 mg.kg^− 1^) based on body weight. Subsequently, attending anesthesiologist evaluated the effect of the initial bolus dose of propofol and followed by a continuous infusion of 250 μg.kg^− 1^.min^− 1^ via an infusion pump to achieve deep sedation [Richmond agitation-sedation scale (RASS) [11] score: − 4] while maintaining spontaneous breathing. During maintenance of sedation, every patient received 200 μg.kg^− 1^.min^− 1^ of propofol, and the infusion rate was adjusted in 25 μg.kg^− 1^.min^− 1^ increments up or down at the discretion of the anesthesiologists in order to maintain deep sedation. [12] If involuntary movement was present, a supplemental dose of propofol (1.0 mg.kg^− 1^) was administered to sustain deep sedation. Patients were observed by means of video cameras for inadvertent movements in the treatment room, and vital sign data were transmitted to a monitor screen in the control room. During sedation, airway interventions were performed when patients demonstrated apnea (> 10 s), oxygen desaturation (SpO_2_ < 90%), and/or airway obstruction, which included checking for equipment malfunctions, airway repositioning, performing the chin lift/jaw thrust maneuver, or establishing bag-mask ventilation as needed. If there was no improvement, other airway devices such as an oral/nasal airway, a laryngeal mask airway (LMA), or an endotracheal tube (ET) were applied. [12] However, if a head mask was used during the PRT, oral airway, LMA, and ET could not be applied, and only nasal airway could be used. Despite management with an airway device, in the case of airway obstruction, treatment was discontinued, and computed tomography (CT) simulation was considered. After the end of PRT, all patients were transferred to a pediatric PACU adjacent to the treatment room, and standard monitoring was continued until the patient was discharged. [13] Recovery was assessed using the RASS score. Awakening time was defined as the time from the end of propofol infusion to the patient displaying alertness and calmness (RASS score: 0).

### Statistical analysis

The primary objective of this study was to evaluate the development of tolerance to propofol during the course of repeated deep sedation for PRT. Tolerance to propofol was determined by statistical significance of the slope by regression analysis of propofol dose over time for every patient. For the primary outcome, the binary logistic regression analysis was performed with potential confounding variables such as age, number of repeated PRT, and an attending anesthesiologist and was adjusted during multivariable analysis. Secondary outcomes included the awakening time, frequency of airway intervention, and sedation-related adverse events. Since treatment was not provided on the weekends, the treatment period was divided into intervals of five days, with an overall number of four weeks. For the descriptive statistics in all patients, all parameters were averaged for each week. An analysis of variance with repeated measures using the mixed model was applied to each parameter, because the numbers of treatments were not the same for all of the patients. To examine the shape of a time-related trend, linear and quadratic equations were applied to the data and tested using contrast analysis. Contrast analysis was used to examine whether the overall significant trend in time was also expressed by a significant difference in the study period. The levels of each session were compared with the level of the first session for each parameter. Continuous variables are expressed as mean ± standard deviation and analyzed by t-test. Categorical variables are expressed as number (percentage) and analyzed by the chi - square test. For the regression analysis, odds ratio with a 95% confidence interval was presented. Analyses were performed using SAS version 9.2 (SAS Institute, Cary, NC, USA), SAS PROC MIXED version 6.1, or SPSS version 20.0 (SPSS Inc., Armonk, NY, USA), and *p* < 0.05 was considered to be statistically significant.

## Results

Sixty-five children were treated with PRT at our institution during the study period. Of these, children who underwent only a simulation session (*n* = 5) and/or those who did not experience sedation with anesthetic agents (*n* = 2) were excluded. All 58 of the enrolled patients (29 males and 29 females) received propofol alone as the sedation anesthetic. The mean (SD) age was 4.5 (2.1) years (Table 1). The mean (SD) number of treatment sessions was 19 (7). The minimal number of PRT was 6 and the maximal number of PRT was 34. Twenty-six children (44.8%) were treated for brain tumors, and the rest were treated for neuroblastoma (*n* = 26) or facial rhabdomyosarcoma (*n* = 6). All PRT procedures were performed with the patient in the supine position.

**Table 1:** Patients’ characteristics

Characteristic	All children (*n* = 58)
Age (years)	4.5 ± 2.1
Height (cm)	102.9 ± 15.0
Weight (kg)	17.4 ± 10.4
Sex (male/female)	29/29
Tumor lesion	
Brain	26 (44.8)
Neuroblastoma, retroperitoneum	16 (27.6)
Neuroblastoma, adrenal gland	8 (13.8)
Neuroblastoma, chest	2 (3.4)
Rhabdomyosarcoma, face	6 (10.3)

Values are mean ± standard deviations or number (%)

**Fig. 1:**
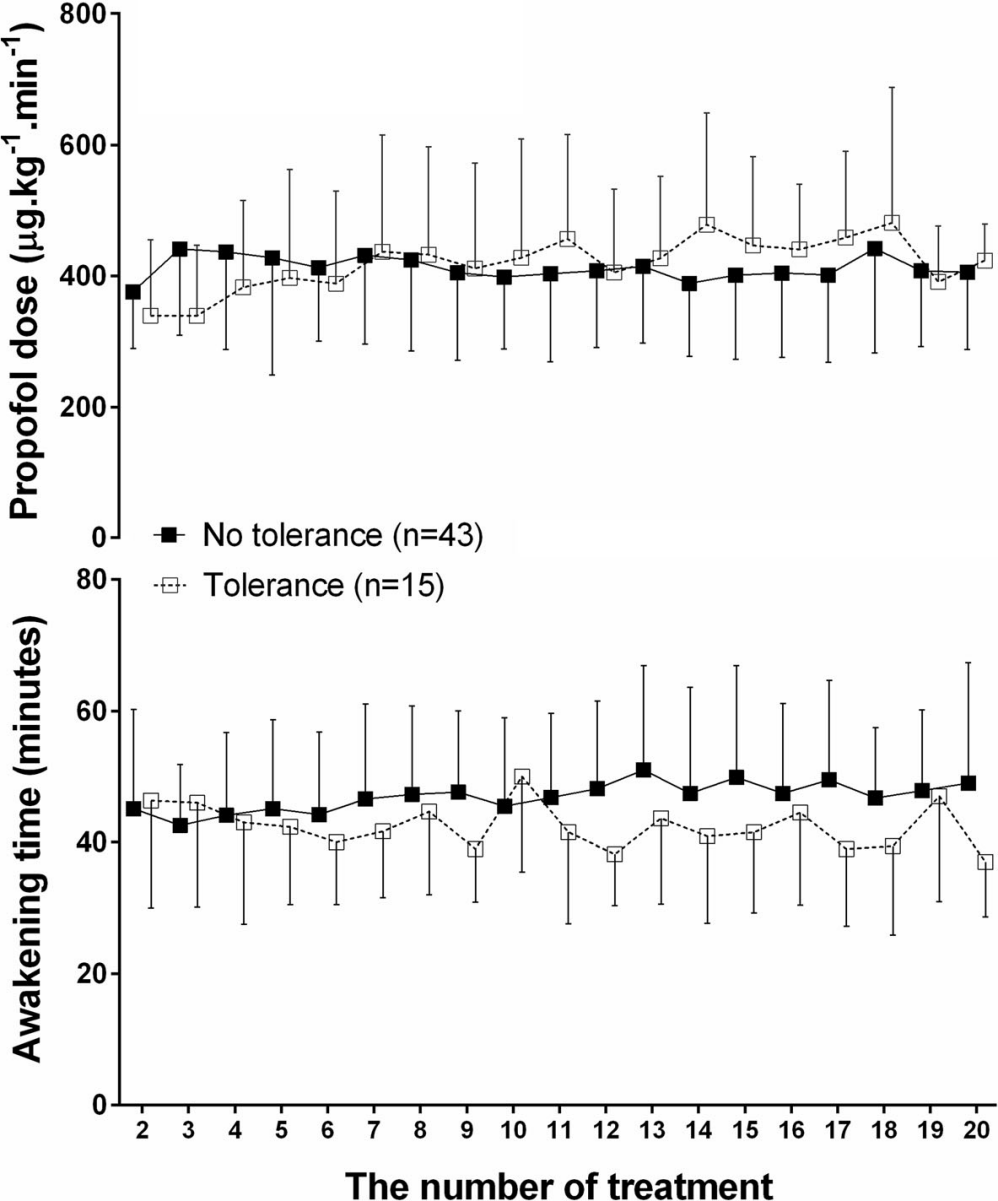
Mean doses of propofol (upper plane) and mean awakening time (lower plane) in children with tolerance to propofol (*n* = 15) and in children without tolerance to propofol (*n* = 43) during the course of proton radiation therapy. Symbols represent the mean and error bars represent standard deviations

Of the 58 patients, 15 patients (26%) developed tolerance to propofol. The proportion of tolerance to propofol was 0.26 and a 95% confidence interval was 0.16 to 0.38. In these 15 patients, the mean (SD) slope of the regression analysis was 0.011 (0.008), indicating that the increase of propofol dosage was minimal. As shown in Fig. 1, there were no significant differences between the children who developed tolerance and the children who did not develop tolerance in mean propofol dose and awakening time over time (*P* = 0.887 and *P* = 0.652, respectively). There were no significant differences in age, height, weight, gender, and the treatment number of PRT between the children who developed tolerance and the children who did not develop tolerance (Table 2).

**Table 2:** Comparison between the children who developed tolerance and the children who did not develop tolerance

	No tolerance (*n* = 43)	Tolerance (*n* = 15)	*p* value
Age (years)	4.4 ± 2.4	4.7 ± 1.3	0.898
Height (cm)	102.5 ± 16.7	104.2 ± 8.4	0.706
Weight (kg)	17.6 ± 11.8	16.6 ± 4.2	0.739
Sex (male/female)	19/24	10/5	0.230
Treatment number of proton therapy	19 ± 6	19 ± 8	0.718

Values are mean ± standard deviations or number

In all patients, there were no significant differences in time-related trend and mean propofol dose over time (*P* = 0.975 and *P* = 0.369, respectively), indicating no constant increase or decrease in the requirement of propofol. Awakening time tended to be long over time (time-related trend *P* < 0.001, linear trend *P* = 0.051, and quadratic trend *P* = 0.266), but overall mean differences in awakening time did not reach a level of significance (*P* = 0.130). Other clinical parameters including hemodynamic variables (e.g., mean arterial blood pressure and heart rate); sedation time; procedure time; and PACU stay time between the various intervals showed no statistically significant differences over time (Table 3).

**Table 3:** Clinical parameters for each week of proton therapy

Parameters	Week 1	Week 2	Week 3	Week 4	*p* value^*^ for mean differences during study period
Mean arterial blood pressure during procedure, mmHg	61.6 ± 9.9	61.0 ± 10.5	61.0 ± 9.9	62.9 ± 10.5	0.655
Mean heart rate during procedure, bpm	90.3 ± 13.9	89.0 ± 13.0	89.6 ± 14.8	86.1 ± 14.5	0.496
Sedation time, min	32.6 ± 17.2	30.2 ± 14.7	28.4 ± 12.6	25.4 ± 12.5	0.361
Procedure time, min	25.3 ± 16.7	23.3 ± 14.4	21.2 ± 12.6	18.7 ± 12.3	0.217
PACU stay time, min	68.3 ± 13.5	70.3 ± 18.6	71.7 ± 18.3	67.8 ± 14.7	0.387

Data are presented as mean ± standard deviations. *PACU,* post-anesthesia care unit. ^*^Analysis of variance with repeated measures using the mixed model

To determine the relationship between the tolerance to propofol and possible confounders, adjust analysis was performed. The results of the univariable analysis indicated that the age, the number of PRT, and attending anesthesiologists was not significantly associated with the occurrence of tolerance to propofol (Table 4).

**Table 4:** Risk of tolerance to propofol among the age, number of treatments, and anesthesiologists

	Univariable analysis	
	OR [95%CI]	P value
Age	1.004 [0.982–1.027]	0.713
Number of treatments	0.994 [0.909–1.087]	0.896
Anesthesiologists		0.681
Anesthesiologists A	1	
Anesthesiologists B	0.556 [0.138–2.238]	
Anesthesiologists C	0.615 [0.139–2.727]	

*CI* confidence interval, *OR* odds ratio

Two patients (3.4%) received a nasal airway from the sixth treatment onwards because of upper airway obstruction exacerbated by steroid treatment. There were no adverse events requiring airway intervention except for additional airway device use. No serious events including aspiration, increased level of care, cardiac arrest, or death occurred during PRT.

## Discussion

In this retrospective study, we demonstrated requirements of propofol were not significantly changed over the PRT period. Also, we did not observe a clear trend in propofol dose over time. Awakening time tended to increase over time, but the overall mean difference was not significant. Thus, based on our findings, we suggest that most of the children undergoing repeated PRT did not develop tolerance to propofol. Although the incidence of 26% tolerance to propofol may still be present, the increase of propofol dosage was minimal and no significant differences in patient characteristics between the children who developed tolerance and the children who did not develop tolerance were found.

Several previous studies have reported that tolerance to propofol does not develop in patients undergoing repeated radiation therapy [1, 14] or electroconvulsive therapy. [15] However, these studies had insufficient data to reach a firm conclusion regarding the nondevelopment of tolerance to propofol. First, the study by Keidan and colleagues [1] did not identify a development of tolerance to propofol during PRT using a bispectral index parameters. However, in their report, the number of patients was too small (*n* = 15) to make this conclusion. The study of Setlock and colleagues [14] also included a small number of children (*n* = 6). Also, propofol was used in conjunction with ketamine or midazolam during PRT, and the authors failed to exclude the possibility of an interaction between propofol and other anesthetics. Third, Soyka and Fischer [15] did not find tolerance to propofol during repeated electroconvulsive therapy. However, they did not consider trends or mean differences in propofol over time: they compared the average doses of propofol at the first session to the last session, rather than comparing all values of propofol during the entire session. In addition, the patients in this study were given one injection at a time; repeated injections given during the same session or prolonged infusion or treatment for a longer period of time could result in either more pronounced or reduced tolerance to propofol. Compared with these previous studies, our study included a larger number of patients (*n* = 58), used propofol alone as a sedative agent, and compared all values of propofol during the entire session. In particular, by determining the tolerance to propofol for every patient, our results suggest the indidence of tolerance and can determine the degree of association between the repeated number of propofol administration and the occurrence of tolerance.

In contrast, one case report in children [16] and one prospective study in adults underwent electroconvulsive therapy [6] suggest that tolerance to propofol may develop with repeated administration, and that increasing doses of the propofol may be required or lead to reduced time to wakefulness over the course of treatment. [6, 8] The demonstration of tolerance relies primarily on the need to increase the amount of medication used to achieve the desired level of sedation, analgesia, or hypnosis. [1] Possible mechanisms of tolerance to propofol can be assumed from the pharmacological evidence for abuse to propofol because the tolerance is one of the diagnostic criteria for abuse. [9] The effect of propofol is mediated by the gamma-aminobutyric acid receptor, which is also known to be potentiated by abusive substances such as alcohol, barbiturates, and benzodiazepines. [17] In addition, propofol increases the extracellular dopamine concentrations by increasing the discharge rate and excitability of dopamine neurons by affecting the mesolimbic reward system. [18] Such evidence might contribute to the development of tolerance to propofol. However, in our study, the amount of propofol was slightly increased in 15 patients with tolerance to propofol, but the required dose was not increased in most patients. In addition, increased awakening time was observed instead of reduced awakening time which is the characteristic of patients who developed tolerance to propofol over the course of PRT. Perhaps this might be due to the repeated daily *nil* per os (NPO) time and sleep cycle changes. [19] Although we administered a bolus of intravenous fluid prior to induction, repeated NPO time for several weeks can be exhausting in young children. As a result, the general condition of the children can worsen and can adversely affect the awakening time. However, none of the previous studies or our study measured the plasma concentrations of propofol to confirm their results. Therefore, it is difficult to make a clear conclusion about this.

Although several studies have reported tolerance to propofol, the reason why the majority of patients did not show tolerance to propofol might be due to the low cumulative time for propofol use. In a previous cost-benefit study comparing the sedation duration of propofol and midazolam reported that tolerance to propofol developed in patients who received propofol over 144 h. [20] In contrast, propofol was given for an average of 9.5 h in our study. Another possible reason is the precise titration of the infusion rate of propofol. Regardless of the agent chosen, tolerance can be delayed with the use of sedation scales to allow for appropriate titration of the infusion. In addition, the use of sedation scales allows for the drip to be adjusted according to the specific patient’s needs so that a minimal amount of the drug is administered. [4]

The use of propofol alone tends to increase the incidence of sedation-related serious adverse events, because it has a dose-dependent response to upper airway collapse by inhibition of the airway dilator muscle and of the upper airway reflexes. [21] However, in our study, although we used propofol alone, the frequency of airway interventions was 2/58 (3.4%). These two children had no specific findings until the fifth treatment, but soft tissue edema was exacerbated by the use of steroid, and airway intervention was subsequently needed. The reason for the low incidence of airway intervention in this study might be due to the fact that all patients underwent optimal airway positioning using an immobilization device such as a base of the skull frame, neck cast, intracranial mask, or a modified Gill-Thomas-Cosman frame during the CT simulation session before performing PRT. Consequently, deep sedation with propofol alone for PRT did not increase the risk of sedation-related serious adverse events.

Our study has several limitations. First, it was a retrospective study; thus, we cannot establish a cause-and-effect relationship in relation to tolerance to propofol. Second, the depth of sedation was assessed using RAAS score by 3 different anesthesiologists. Thus, it could increase the risk of inter-observer bias. However, all of them were experts in pediatric sedation for more than 4 years and the propofol dose determined by the RASS score were not significantly associated with the development of the tolerance depending on 3 different anesthesiologists. Third, the incidence of 26% tolerance to propofol was observed, but since the plasma concentration of propofol was not measured, it cannot be confirmed whether the tolerance to propofol actually developed. Thus, further prospective studies are recommended including more objective methods of assessing deep sedation and measurement of plasma concentrations of propofol to determine the occurrence of tolerance.

## Conclusions

We found that 74% of patients did not develop tolerance after repeated deep sedation using propofol infusion over several weeks. Although the incidence of 26% tolerance to propofol may still be present, the increase in propofol dose was minimal. Therefore, the use of repeated propofol for children was safe. Further large-scale prospective studies are needed before clinical relevance can be defined.

## Abbreviations

CT: Computed tomography
ET: Endotracheal tube
LMA: Laryngeal mask airway
NPO: Nil per os
PACU: Post-anesthesia care unit
PRT: Proton radiation therapy
RASS: Richmond agitation sedation scale
